# 186 Youth-Physical Activity Towards Health in Northern Ireland (Y-PATH NI) Study: Attitudes towards and experiences of physical activity and physical education

**DOI:** 10.1093/eurpub/ckae114.125

**Published:** 2024-09-26

**Authors:** Angela Carlin, Julia McClelland, Alison Gallagher, Sarahjane Belton, Wesley O’Brien, Marie Murphy

**Affiliations:** Centre for Exercise Medicine, Physical Activity and Health, Sports and Exercise Sciences Research Institute, Ulster University, Belfast, United Kingdom; Centre for Exercise Medicine, Physical Activity and Health, Sports and Exercise Sciences Research Institute, Ulster University, Belfast, United Kingdom; Nutrition Innovation Centre for Food and Health (NICHE), Biomedical Sciences Research Institute, Ulster University, Coleraine, United Kingdom; School of Health and Human Performance, Dublin City University, Dublin, Ireland; School of Education, University College Cork, Cork, Ireland; Centre for Exercise Medicine, Physical Activity and Health, Sports and Exercise Sciences Research Institute, Ulster University, Belfast, United Kingdom; Physical Activity for Health Research Centre (PHARC), Institute for Sport, Physical Education and Health Sciences, University of Edinburgh, Edinburgh, Scotland

## Abstract

**Purpose:**

Approximately 20% of post-primary school pupils in Northern Ireland (NI) receive the recommended 120 minutes of physical education (PE) per week. A more detailed understanding of NI schoolchildren’s perceptions of context specific physical activity, the participation barriers they face in relation to PE, in addition to factors that support them to lead a physically active lifestyle is key in understanding how best to encourage physical activity participation in this population. The purpose of this study was to assess current physical activity levels and explore the attitudes and experiences of PE amongst adolescents in NI.

**Methods:**

A mixed-methods approach was used. A cross-sectional online survey was completed by a sample of NI schoolchildren in Years 8-10 (n = 1541) in 2023. The survey consisted of validated scales and study specific items. Focus groups (n = 17) were conducted with pupils in Years 8-12. Descriptive statistics and chi-square analyses were used to compare differences across genders and year groups. Qualitative data were analysed using thematic analysis, data were summarised, and themes were generated.

**Results:**

One-fifth of participants reported meeting the physical activity guidelines, with differences observed for gender (male = 31% vs female = 14%) and year group (Year 8 = 24% vs Year 10 = 17%). Males tended to report higher levels of enjoyment of PE than females. The main barriers to participation in PE identified through focus group discussions included getting changed to take part in PE and not enjoying some of the activities offered. Participants liked participating in PE with their peers, enjoyed learning new skills, and found PE teachers to be supportive. In terms of how to enhance provision of PE in schools, participants reported wanting more variety and to be given a choice of activities in their PE classes.

**Conclusions:**

The findings from this study suggest that there are barriers to participation in PE classes. These findings will help policymakers and researchers develop interventions that will be effective in enhancing PE provision with post-primary schools and increasing physical activity levels amongst youth.

**Support/Funding Source:**

This study is funded by Northern Ireland Chest Heart and Stroke.

